# Solitary ulcer at the mons pubis in a heterosexual male patient

**DOI:** 10.1007/s10354-026-01159-2

**Published:** 2026-05-11

**Authors:** Pia Hajsek, Teresa Kränke, Birgit Sadoghi

**Affiliations:** Department of Dermatology and Venereology, Medical University of Graz, Auenbruggerplatz 8, 8026 Graz, Austria

**Keywords:** Syphilis, Genital ulcer, Sexually transmitted infection, Syphilis, Genitalulkus, Sexuell übertragbare Infektion

## Abstract

**Background:**

Syphilis is a curable, systemic sexually transmitted infection caused by the bacterium *Treponema pallidum *subsp*. pallidum* (TP). It is known as the “great imitator” due to its potential to mimic various dermatoses, possibly delaying correct diagnosis, particularly if syphilitic lesions appear in atypical locations.

**Case description:**

We report the case of a 54-year-old heterosexual white man who presented with a solitary, indurated ulcer on the mons pubis. Patient history revealed minimal trauma to the area 3 weeks prior, resulting from shaving. Initial topical treatment for bacterial folliculitis resulted in worsening of the lesion. Finally, in site polymerase chain reaction (PCR) for TP and syphilis serology verified the diagnosis of a chancre.

**Conclusion:**

This case underlines the importance of including syphilis in the differential diagnosis of any anogenital ulceration, even in uncommon anatomical sites and populations, thereby enabling prompt diagnosis and treatment.

## Abstract (de)

**Hintergrund:**

Syphilis ist eine systemische, heilbare, sexuell übertragbare Infektion, die durch das Bakterium *Treponema pallidum *subsp*. pallidum* (TP) verursacht wird. Die Erkrankung wird als „großer Imitator“ bezeichnet, da sie häufig verschiedene Dermatosen imitieren kann. Folglich ist eine adäquate Diagnosestellung oftmals verzögert, insbesondere, wenn syphilitische Läsionen an atypischen Stellen auftreten.

**Fallbeschreibung:**

Wir präsentieren den Fall eines 54-jährigen heterosexuellen Mannes, der sich mit einem solitären, indurierten Ulkus im Bereich des Mons pubis vorstellte. Die Anamnese ergab ein minimales lokales Trauma etwa 3 Wochen zuvor aufgrund einer durchgeführten Rasur. Eine initiale topische Behandlung bei Verdacht auf bakterielle Follikulitis führte zu einer Progredienz der klinischen Symptome. Abschließend bestätigte sich mittels Polymerasekettenreaktion (PCR) auf TP und einer reaktiven Syphilisserologie die Diagnose einer primären Syphilis (harter Schanker).

**Schlussfolgerung:**

Dieser Fall unterstreicht die Notwendigkeit, eine Syphilis als Differenzialdiagnose jeglicher anogenitaler Ulzerationen in Betracht zu ziehen, selbst an ungewöhnlichen anatomischen Lokalisationen und nicht nur in bekannten Risikogruppen, um eine korrekte Diagnose und Therapie zeitnah zu ermöglichen.

## Case presentation

A 54-year-old heterosexual white man presented directly to the emergency dermatology outpatient clinic due to a solitary erosion with elevated edges on the mons pubis, first noticed by the patient 3 weeks earlier. The patient presented for the first medical evaluation after a failed attempt to self-treat with over-the-counter antiseptics. He described an uncomfortable sensation at the site of the lesion without severe pain. He reported a minor abrasion after shaving of the area, and he denied fever, chills, or any other systemic symptoms.

His past medical history included hypertension, managed occasionally with candesartan/amlodipine.

On clinical examination, a solitary thumb-nail-sized erosion with an indurated base and edges was observed on the mons pubis. No regional lymphadenopathy or any other cutaneous involvement was observed. Based on the reported history of minor skin trauma and clinical presentation, indurated folliculitis was suspected and appropriate treatment was initiated with an antiseptic compress and topical application of natriumfusidat ointment.

**Fig. 1:**
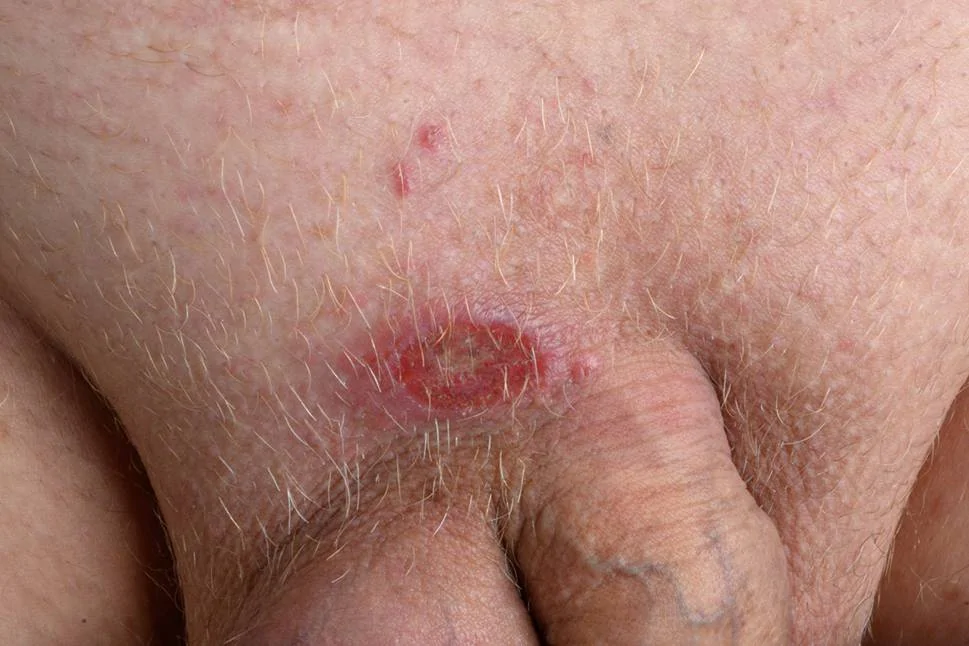
Solitary ulcerous lesion at the mons pubis

The patient returned to the emergency dermatology outpatient clinic 10 days later due to lack of improvement. The erosion had progressed to an ulcer, but appeared unchanged in size (Fig. 1). He was referred to the sexually transmitted disease outpatient clinic, where a detailed sexual history was obtained. He reported that his last unprotected sexual contact was with a casual female acquaintance about 2 weeks before appearance of the lesion and he identified as heterosexual. The patient could not specify the exact number of sexual partners in the preceding 6 months. A history of a previous *Chlamydia trachomatis* infection and possible gonorrhea infection was reported by the patient.

What is your diagnosis?

A. Recurrent herpes simplex virus infection
B. Squamous cell carcinoma
C. *Primary syphilis*
D. Atypical deep mycobacterial infection

Polymerase chain reaction of ulcer material revealed *Treponema pallidum *(TP). Syphilis serology was reactive, with rapid plasma reagin (RPR) testing yielding a titer of 1:4. Immunoblot testing showed reactive TP-specific immunoglobulin (Ig)G and IgM antibodies. Based on the clinical picture and laboratory results, the diagnosis of a chancre (primary syphilis) was made.

Guideline-based therapy was initiated, and the patient was treated with a single intramuscular gluteal injection of 2.4 million units of benzathine penicillin G. The patient was told to abstain from sexual activity until complete healing of the lesion and to notify all sexual partners from the 3 months prior to symptom onset. At the first follow-up visit 1 month later, the ulcer had re-epithelialized and post-inflammatory hyperpigmentation was visible.

## Discussion

### Etiology, pathogenesis, and epidemiology

Syphilis, a disease that runs in stages, is a sexually transmitted infection (STI) caused by the spirochete bacteria TP. Infection occurs at the site of inoculation, mainly via microscopic defects in the skin or mucous membranes. Moreover, the bacterium is able to cross highly selective barriers, including the placental, retinal, and blood–brain barriers. Rapid systemic spread via both the vascular and lymphatic systems may lead to organ manifestation, even in early stages [1–3].

According to the latest Annual Epidemiological Report for 2023, published by the European Centre of Disease Control and Prevention (ECDC), the number of reported cases of syphilis is increasing significantly, with a 13% increase in overall notification rate compared to the previous year and a 100% increase compared to 2014. Although the highest incidence rates of syphilis are reported among men who have sex with men, the epidemiological trend is not confined to this population. Syphilis is no longer restricted to traditionally defined high-risk populations [4]. Testing for TP should therefore not be limited to individuals with a classic risk profile.

### Syphilis stages

The patient’s clinical presentation with a solitary, painless, indurated ulcer and unprotected sexual intercourse was strongly suggestive of primary syphilis, and the lesion was consistent with a chancre.

Establishing the correct diagnosis at the first presentation is particularly challenging in such clinical scenarios. Initial assessments rely heavily on patient-reported history, which can be incomplete, unintentionally inaccurate, or occasionally intentionally misleading. Patients may attribute symptoms to seemingly plausible but unrelated events—such as minor trauma or shaving-related abrasions—especially when these precede lesion development temporally. Such assumptions, while understandable, can lead to significant bias.

Additionally regional lymphadenopathy can be present in primary syphilis, although it was absent in this case [2, 3, 5, 6]. Lesions typically develop at the site of inoculation, as demonstrated in this case, and are consequently most common in the genital area. However, extragenital presentation may occur [6].

If the patient had not received treatment, secondary syphilis would have developed within a few weeks. This would typically present as a non-pruritic macular or papular rash involving mainly the trunk. Systemic symptoms, such as fatigue, mild fever, and systemic lymphadenopathy, are not rare. Other disease-specific lesions that can be observed are opalescent mucous patches, moth-eaten alopecia, clavi syphilitic, and condylomata lata [2, 3, 5, 6]. Organ manifestations and central nervous system involvement are also possible [3].

### Diagnosis

Syphilis is diagnosed combining clinical findings, direct detection methods, and serology. The diagnostic algorithm followed included a positive polyvalent screening test for syphilis, followed by an RPR titer of 1:4. The results were confirmed with an immunoblot, which presented reactive TP-specific IgM and IgG antibodies (Tp47, TmpA, Tp17, Tp15). Additionally direct detection of TP using PCR was performed due to the presence of an eroded lesion [3, 5, 7]. According to the steep rise in STI cases, we strongly recommend that all patients presenting with anogenital ulcerations—particularly painless lesions—undergo diagnostic evaluation for TP, regardless of their reported sexual history. As mentioned earlier, sexual history may be incomplete, or inaccurately reported, for various reasons. A low diagnostic threshold is warranted to enable timely treatment and to prevent further transmission.

### Therapeutic management

A depot penicillin is the first-line treatment for syphilis at all disease stages and patient populations. For the treatment of primary, secondary, and early latent syphilis, all guidelines recommend a single intramuscular dose of benzathine penicillin G (2.4 million IE), which was accordingly administered in our case. Late latent syphilis, syphilis of unknown duration, and tertiary syphilis are treated with three doses of 2.4. million IE benzathine penicillin G for three consecutive weeks [3, 5].

## Conclusion

Syphilis often mimics other dermatoses and the authors emphasize the recommendation to include *Treponema pallidum* in the differential diagnosis of every anogenital erosion and ulceration. Atypical presentations, including changes in typical morphology, number, or anatomical location, are possible, making the rapid diagnosis of syphilis a constant challenge for physicians [2, 5].

## Data Availability

The data of this article are available on request by the corresponding author.
